# *In vitro* Models and On-Chip Systems: Biomaterial Interaction Studies With Tissues Generated Using Lung Epithelial and Liver Metabolic Cell Lines

**DOI:** 10.3389/fbioe.2018.00120

**Published:** 2018-09-03

**Authors:** Milica Nikolic, Tijana Sustersic, Nenad Filipovic

**Affiliations:** ^1^Faculty of Engineering, University of Kragujevac, Kragujevac, Serbia; ^2^Steinbeis Advanced Risk Technologies Institute doo Kragujevac, Kragujevac, Serbia; ^3^Bioengineering Research and Development Center, Kragujevac, Serbia

**Keywords:** Calu-3, HepG2, *in vitro* models, organ-on-a-chip, epithelial barrier, toxicology

## Abstract

*In vitro* models are very important in medicine and biology, because they provide an insight into cells' and microorganisms' behavior. Since these cells and microorganisms are isolated from their natural environment, these models may not completely or precisely predict the effects on the entire organism. Improvement in this area is secured by organ-on-a-chip development. The organ-on-a-chip assumes cells cultured in a microfluidic chip. The chip simulates bioactivities, mechanics and physiological behavior of organs or organ systems, generating artificial organs in that way. There are several cell lines used so far for each tested artificial organ. For lungs, mostly used cell lines are 16HBE, A549, Calu-3, NHBE, while mostly used cell lines for liver are HepG2, Hep 3B, TPH1, etc. In this paper, state of the art for lung and liver organ-on-a-chip is presented, together with the established *in vitro* testing on lung and liver cell lines, with the emphasis on Calu-3 (for lung cell lines) and Hep-G2 (for liver cell lines). Primary focus in this review is to discuss different researches on the topics of lung and liver cell line models, approaches in determining fate and transport, cell partitioning, cell growth and division, as well as cell dynamics, meaning toxicity and effects. The review is finalized with current research gaps and problems, stating potential future developments in the field.

## Introduction

*In vitro* models are the starting point in biological and medical research. With scientific progress and emergence of different *in vitro* models, knowledge of the entire organism behavior is growing. Together with experimental *in vitro* models, computer *in silico* models are being developed. Different types of models are developed, at different scales—macro, meso, micro, nano, depending on whether they explain the behavior of the whole system (macro; finite element models), behavior at the level of molecular clusters (meso; dissipative particle dynamics models) or behavior at the molecular level (micro, nano; molecular dynamics models). Results obtained from *in vitro* and *in silico* models should be compared and verified. The final goal is to develop adequate *in silico* models, which reduce costs and time of experimental measurements and provide satisfactory results. However, first we need the results of *in vitro* models. *In vitro* models were used for many segments of the human organism—for blood-brain barrier (Ogunshola, 2011), the study of osteoarthritis (Johnson et al., 2016), psoriasis (Jean and Pouliot, 2010), myocardial tissue (Vunjak Novakovic et al., 2014) and myocardial ischemic injury (Tumiati et al., 1994) and paroxysmal supraventricular tachycardia (Wit et al., 1971), murine middle ear epithelium (Mulay et al., 2016), Alzheimer's disease (Stoppelkamp et al., 2011), thrombosis (Zhang et al., 2017) and vascular inflammation (Ahluwalia et al., 2018), as well as for different models of cancer (Katt et al., 2016), etc.

Calu-3 cell line was established in 1975 from a metastatic site (pleural effusion) in a 25-year-old Caucasian male with lung adenocarcinoma (Memorial Sloan Kettering Cancer Center, 2018). Current research shows that this cell line possesses characteristics similar to primary epithelial cells and can be used for investigation of the airway epithelial barrier to evaluate the regularity and irregularity of the barrier functions. Formed epithelial barrier can be used for investigation of diseases and for testing novel therapies and medicaments.

Although known for being part of the digestive tract and for the role of metabolizing xenobiotics and nutrients (carbohydrates and lipids), the liver is involved in more than 300 vital functions (Angier, 2017). When it comes to toxicological studies, chemical of interest is often tested on the liver. In recent years, new *in vitro* and *in silico* technologies have enabled insight into toxic mechanisms in order to replace or reduce the use of animals in tissue examinations (Gubbels-van Hal et al., 2005; Jie et al., 2016; Comenges et al., 2017). This is done at the molecular level to understand how changes at lower levels influence higher levels of biological organization (e.g., tissue, organs, etc.; EPA, 2003). One of the cell lines that has recently gained considerable attention is human hepatocarcinoma cell line, HepG2, that is used in *in vitro* studies on liver tissue (Gonzales et al., 2015). It has one nucleus and the epithelial-like morphology (Wilkening et al., 2003). HepG2 cell line is originally extracted from a 15-year-old Caucasian boy in the form of hepatocellular carcinoma (Gonzales et al., 2015). However, many of the mechanisms associated with the normal human hepatocytes are to be found in HepG2 cells, some of which are plasma proteins secretion, bile acids production, as well as detoxification processes. It is also reported that hepatoma cells possess receptors for insulin, transferrin, estrogen and low-density lipoproteins (Bouma et al., 1989; Gonzales et al., 2015), which means that they have the detoxification mechanisms, performed by rendering biotransformation reactions (Dehn et al., 2004).

The following section presents *in vitro* models for lung and liver cell lines. After that, data processing and available models of lung and liver cell lines are discussed. The fourth section contains on-chip review related to the previously mentioned cell lines, and, finally, the conclusion section provides a summary of the state-of-the-art and critically observes possible future steps in this field.

## Retrospective of *in vitro* models of lung and liver cells

### Modeling of lung using calu-3 epithelial cell lines

Even though Calu-3 cells represent immortalized cells, they still possess many characteristics of primary airway cells and can be used for observation of transport, metabolism and testing novel medicament approach (Zhu et al., 2010).

**Figure 1:**
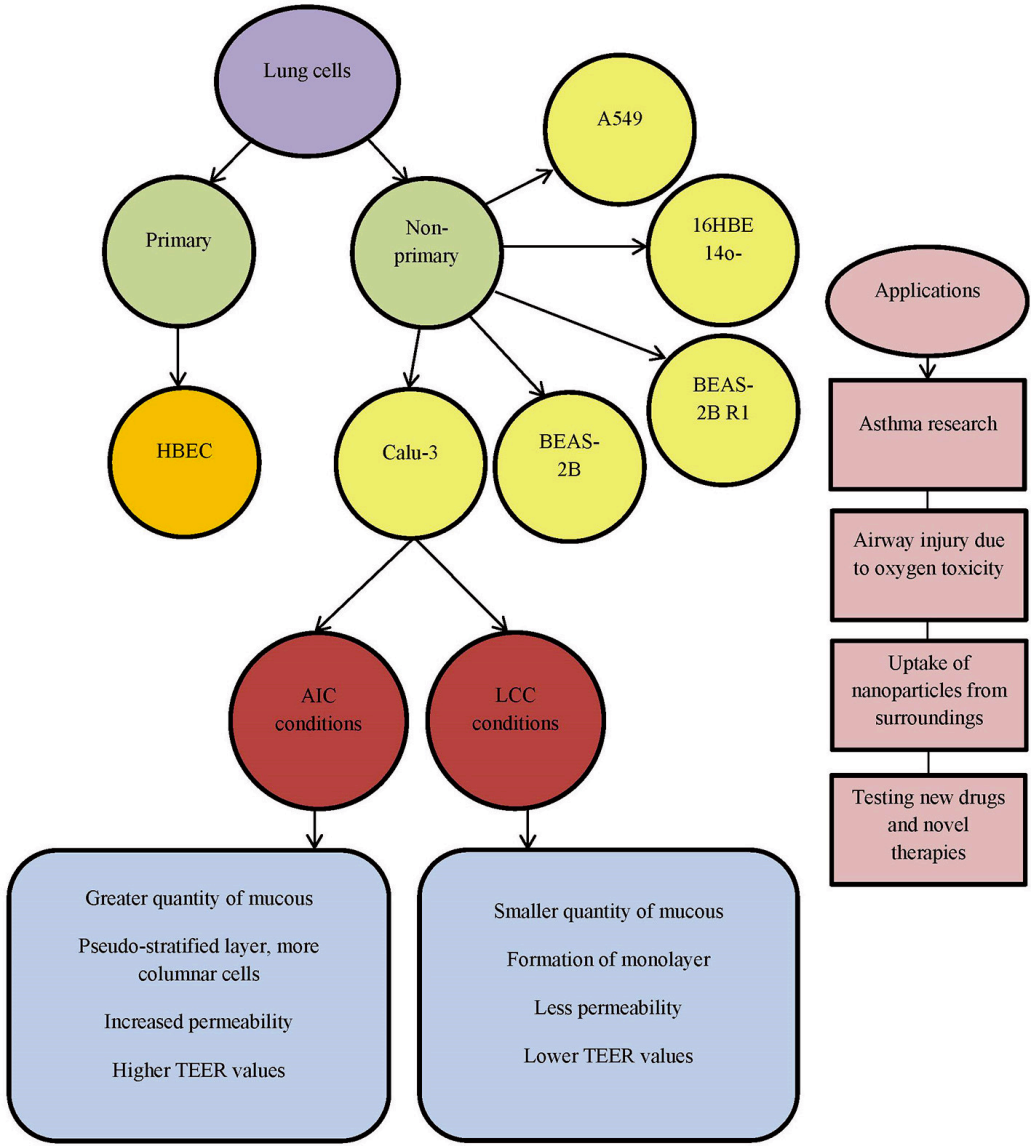
List of commonly used lung cell lines. AIC, air-interfaced culture; LCC, liquid-covered culture.

Differentiated human bronchial epithelial cell culture systems were evaluated for asthma research —primary cells (human bronchial epithelial cells, HBEC) and non-primary cells, cell lines [Calu-3, BEAS-2B, BEAS-2B R1; (Stewart et al., 2012)] (Figure 1). More physiological models can be obtained with HBECs cultured at air-liquid interface with specifically determined medium which differentiates appropriate phenotype. Cells possess the ability to differentiate into goblet (MUC5AC+), to form ciliated layer (β-tubulin IV+) and to develop high transepithelial electric resistance (TEER), which serves as an indirect measure of occurrence of tight junctions (ZO-1 protein) and as a marker of disruption of the epithelial layer. Calu-3 cells grow up to confluence within 5 days and form tight epithelial monolayer over 21 days in culture. Cell layer tightness is confirmed with TEER measurements. Permeability can be observed using flu-Na uptake and the current results show that there is connection between TEER parameter and flu-Na uptake and membrane permeability (Haghi et al., 2010). Cell surface P-gp expression on Calu-3 is time dependent and does not depend on the cell passages. It was concluded that mucous secretion increases with time in culture. Determination of mucous quantity can be done with alcian blue. Cells differentiation can be evaluated by confocal imaging and qPCR (Stewart et al., 2012). Calu-3 cells take the greatest amount of time to become fully confluent, but more homogenous with increased mucus secretion. Grainger et al. (2006) in their research also concluded that Calu-3 cell line could be used to model the function and behavior of airway epithelial barrier. They cultured cells as air-interfaced culture (AIC) and as liquid-covered culture (LCC). In comparison to environmental conditions AIC provided greater quantity of mucus covering the cell surface, pseudostratified layer with more columnar cells (LCC creates monolayer)and more permeable cells. By comparing AIC and LCC, authors concluded that Calu-3 cell line cultured with AIC produced cell layer more similar to real airway epithelial morphology and electrical resistance *in vivo*, than cells cultured using LCC (Figure 1). Kreft et al. came to similar conclusion (Kreft et al., 2015) by investigating Calu-3 cell line under different culture conditions to ensure optimized *in vitro* model to investigate bronchial epithelial function. Calu-3 cells were tested in A-MEM media at air-liquid interface (A-L) and at liquid-liquid interface (L-L). A-L interface showed to be more natural in physiological way, forming pseudostratified columnar epithelium with more microvilli and secretory vesicles, showing higher TEER values and lower permeability of dextran. Longer time in culture significantly decreased dextran permeability and increased expression of drug transporter. Major conclusion from this investigation was that cell culture conditions as well as time in culture affect cell differentiation, barrier function, permeability values and drug transporter expression. All these should be standardized for Calu-3 cell line as an *in vitro* model for drug delivery system and lung diseases.

Zhu et al. gave a review on human cultured airway epithelial cells, Calu-3 (Zhu et al., 2010). They used a model for assessing the effects of oxygen concentration, positive airway pressure and certain pharmacological agents. This cell line proved to be useful for studying the respiratory diseases, airway injury related to oxygen toxicity and evaluation of novel therapies (Figure 1). Isolated Calu-3 cells can mainly mimic airway epithelial cells, but absence of systematic inflammation should not be neglected in certain investigations. Another important characteristic in studying lung diseases is the uptake of nanoparticles from the surroundings, polluted air and water. Especially dangerous are carbon nanoparticles (CNP, beads). CNP uptake in the body can occur through inhalation of nanoparticles from air and can cause lung disease or further enter into circulatory system and even brain (Banga et al., 2012). Carbon nanoparticles affect barrier function of the airway epithelial Calu-3 monolayer by reducing it. Loss of cells viability decreases TEER, measured on the cellular monolayer, which is related to cytokine release. Calu-3 cell line can be used for observing respiratory irritation and testing toxicity of drugs and their medium by using MTT assay (Ihekwereme et al., 2014). Epithelial cell lines, like Calu-3, can be used for testing new drugs and novel therapies. Ong et al. focused on pharmaceutical applications of the Calu-3 cell line (Ong et al., 2013), but analyzed the use of other cell lines, such as bronchial cell lines 16HBE14o-, BEAS-2B and alveolar cell line A549. Their conclusion based upon the literature is that AIC has advantage over LCC surroundings and that TEER can be used as a measurement of integrity of the formed monolayer. Drug transport is based on layer permeability. Calu-3 cells show expression and functional activity of P-glycoprotein (P-gp), the multidrug resistance associated proteins (MRPs), as well as breast cancer resistance protein (BCRP). These transporters are detectable in human lungs, *in vivo*, and in Calu-3 cell line. Drug-drug interactions could result in increased toxicity and side effects to diverse therapeutic outcomes. For example, Mamlouk et al. showed that nonsteroidal anti-inflammatory drugs (aspirin, ibuprofen, etc.) reduced the uptake of salbutamol across Calu-3 cells (Mamlouk et al., 2013). Calu-3 cell line is also used in investigation of anticancer therapeutics for growth inhibition, due to its cancer origin (Ong et al., 2013).

### Modeling of liver using HepG2 metabolic cell lines

**Figure 2:**
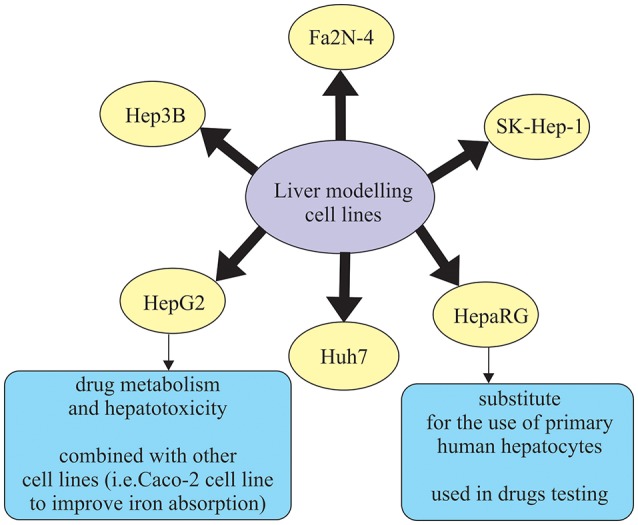
List of commonly used liver cell lines.

Human hepatic cell lines, no matter if they are cancer-derived or immortalized hepatocytes, can be effectively cultured and used for different purposes. Some of the widely used hepatic cell lines are HepG2, Huh7, Hep3B, and SK-Hep-1, which are derived from hepatocellular carcinoma (HCC), and HepaRG, which is an HCC cell line that includes both hepatocytes and biliary-like cells (Guo et al., 2011; Gerets et al., 2012) (Figure 2). However, human hepatic cell lines have some limitations such as lower and variable cytochrome P450 enzymes (CYP450) expression presence which is not the case in primary human hepatocytes (Guo et al., 2011). In that sense, the results obtained with induction of CYP450 enzymes are more promising, meaning that drug transporter MDR1 is better in a novel hepatic cell line, Fa2N-4 (Mills et al., 2004). Another cell line, HepaRG cell line, is a promising substitute for primary human hepatocytes. It was noticed that in the case of low density seeding, HepaRG cells proliferate and differentiate to confluence, only to form colonies of hepatocytes. These colonies are than surrounded by biliary epithelial cells that show CYP450 expression levels, which is the result comparable to primary human hepatocytes (Guillouzo et al., 2007). What is more interesting, regarding drug delivery, is the ability of HepaRG cells to identify drugs that are likely to induce liver injury (Tomida et al., 2015). Additionally, HepaRG cells also show a response that is more robust to the inflammatory stimuli (e.g., IL-6, TNFα), in comparison to the primary human hepatocytes, which could be a result of the genetic variation (Klein et al., 2015) (Figure 2).

In recent years, lot of advancements have been made in the area of culture systems, which have enhanced functionality and stability of liver cells *in vitro* (Zeilinger et al., 2016). In order to describe the behavior of the native organ *in vivo*, cell types used in hepatic *in vitro* research have to fulfill some of those functions, depending on the study aim (Zeilinger et al., 2016). Many different *in vitro* liver models have been designed in order to accurately describe chemical treatment that will be able to be translated to *in vivo* responses. Out of the proposed methods, those that remain applicable for *in vitro* liver toxicity testing are liver slices, cell lines, and primary hepatocytes (Soldatow et al., 2013). Liver tissue slices are used because they retain liver structure, have good correlation *in vitro*/*in vivo* (because they contain cell types found *in vivo*) and maintain zone-specific cytochrome activity and mechanisms of toxicity (Lerche-Langrand and Toutain, 2000). Studies using tissue slices are typically in culture length that ranges from 30 min to 5 days and follow the parameters like oxygen tension, media and supplements, and culture system (i.e., shaken flasks, multiwall plates, stirred wells, etc.). They are usually modified to increase cell viability and reduce degenerative changes that are present in the tissue during the examination (Soldatow et al., 2013). Immortalized cell lines derived from the liver mostly do not possess phenotypic characteristics of the liver tissue (Yu et al., 2001). Most commonly used immortalized liver-derived cell lines are Fa2N-4, HepG2, Hep3B, PLc/PRFs Huh7, HBG, and HepaRG (Guguen-Guillouzo and Guillouzo, 2010; Guguen-Guillouzo et al., 2010) (Figure 2). Primary hepatocytes and hepatocyte-like cells have restricted application using standard culture conditions for some types of toxicity testing due to the problems in long-term maintenance of their functionality, lack of proper absorption, distribution, metabolism, and excretion (ADME) properties (Soldatow et al., 2013). Primary hepatocytes suspensions are also widely used in moderately high-throughput toxicity studies (Soldatow et al., 2013). Previous studies demonstrated that suspensions also keep high levels of functionality, which enables better correlation to *in vivo* toxicity studies (O'Brien and Siraki, 2005; Hewitt et al., 2007). For example, Griffin and Houston (2005) concluded that suspensions of hepatocytes allow for better and more accurate prediction of internal clearance rate in comparison to the conventional monolayer cultures. However, in *in vitro* testing, primary hepatocyte cultures have been mostly used, as they are able to maintain functional activities for 24–72 h. Therefore they can be applied not only in studies for enzyme induction and inhibition, allowing for medium-throughput monitoring of compounds, but are also ideal for studying interspecies and inter-individual differences in metabolism (LeCluyse, 2001; Hewitt et al., 2007). Primary hepatocyte cultures are often combined with inflammatory mediators. The reason for this is that traditional 2D hepatocyte cultures are not well applicable in high-throughput screening (Soldatow et al., 2013). An *in vitro* combination—drug/inflammatory cytokine/inflammatory mediator co-treatment was used in the study by Cosgrove et al. (2009) to reproduce clinical drug hepatotoxicity. It was performed especially for idiosyncratic drugs, not only in cultured primary human and rat hepatocytes, but also in HepG2 cells.

Additionally, *in vitro* studies often combine two cell lines. For example, one study by Scheers et al. (2014) combined well established Caco-2 cell line with human liver cells HepG2 in order to improve iron absorption. The results show that this approach is a possible alternative to the traditional Caco-2 *in vitro* model for iron absorption (Scheers et al., 2014).

Main applications of liver cell lines used in *in vitro* research are in cancer development studies and therapy (Zeilinger et al., 2016). Cell line HepG2, as tumor cell line, has been investigated for the purposes of examining specific metabolic pathways that are related to liver tumors or testing development of drugs for cancer therapy. It should be emphasized that usually different sensitivities in various tumor cell lines from different origins are examined in parallel, in order to cover different types of cancer. For example, HepG2 cells were used in a research that analyzed the expression and regulation of cancer-related transcription factors (Samatiwat et al., 2016). HepG2 cell line can also be used to examine drug metabolism and hepatotoxicity. This is the case in the study by Palabiyik et al. (2016), who investigated the drug metabolism and hepatotoxicity, more specifically acetaminophen toxicity and prevention. HepaRG cell line also shows a promising alternative for the use of primary human hepatocytes (PHH) for the studies on drug metabolism, disposition, and toxicity (Lübberstedt et al., 2011; Andersson et al., 2012). They showed that HepaRG cells have the ability to highly differentiate and express typical hepatic functions. It means they could be used in studies including CYP-dependent metabolism, CYP induction, and drug transporter expression (Andersson et al., 2012). HepaRG cells, when used in drug toxicity tests, demonstrate similar response to the effects of acetaminophen as PHH and higher activation of genes related to liver damage in comparison to HepG2 cells. On the other hand, they show reduced sensitivity to the detection of hepatotoxic drugs (Gerets et al., 2012). As a result, it can be concluded that HepaRG cells can be an alternative to PHH in screening studies for CYP induction (Zeilinger et al., 2016).

## Developed methods for liver and lung cells analysis—toxicology assessment, cell growth and division

Computer models and mathematical models of the lungs are rare. There are no many reports in literature related to this subject. Most of the developed models for lung-on-a-chip microfluidic device include imaging methods for 3D cell biology, like confocal laser scanning microscopy, two-photon and multiphoton microscopy, transmission and scanning electron microscopy, time-lapse imaging (Konar et al., 2016). Konar et al. analyzed applications of lung organoid, which assumes 3D tissue-engineered culture system that accurately replicates the histological and functional aspects of the *in vivo* tissue. Application of such system can be divided into several areas: cancer research, inflammations and infections of lungs, drug toxicity testing and drug development and finally personalized medicine.

A model of the lung-on-a-chip was developed by Hancock and Elabbasi (2018) with COMSOL Multiphysics software, according to the model presented by Huh (2015). COMSOL ensures methods for simulating fluid-structure interaction, nonlinear structural materials, laminar fluid flow, dilute species transport and particle tracing capability. The PDMS membrane and the walls can be modeled as neo-Hookean, Mooney-Rivlin or Ogden nonlinear material models. Additional features may be added, such as simulation of drug and nutrient transport, uptake by cells on the porous membrane, etc. COMSOL model provides insight into device functioning in dependence on the manufacturing and chosen material.

Savla et al. (2004) mathematically modeled airway epithelial wound closure during mechanical cycling strain. Modeling part was based upon *in vitro* model of human and cat airway epithelial cells (AEC) cultured cells, used for studying the repair mechanisms of wounded airway epithelial monolayer subjected to cyclic strain. Mathematical representation of the model included extended diffusion equation, combining parameters such as diffusion coefficient, spreading coefficient, and proliferation coefficient. These parameters influence wound closure and concentration of the cells to a large extent.

One of the goals in the future will be improvement of the mathematical and computer models of lung-on-a-chip device, as well as automation of the imaging techniques.

On the other hand, computer liver models were developed mainly with the goal to analyse toxicity of drug metabolites. Some studies used multi-scale approaches, such as physiologically-based kinetic/dynamic (PBK/D) models that describe the transportation of the chemicals in the body (Sala Benito et al., 2017). These models were often combined with some refinements (Gubbels-van Hal et al., 2005) to estimate the bioavailability and partitioning of the chemical in the assay in order to improve the estimations of concentrations *in vivo*, by using extrapolations of *in vitro* obtained concentrations (Sala Benito et al., 2017). This modeling approach is useful in the chemical risk assessment, when prioritization of chemical testing is important (Kramer et al., 2015; Bell et al., 2017). Whenever possible, *in vitro* studies were combined with *in vivo* studies in order to validate the proposed approach (Comenges et al., 2017).

**Figure 3:**
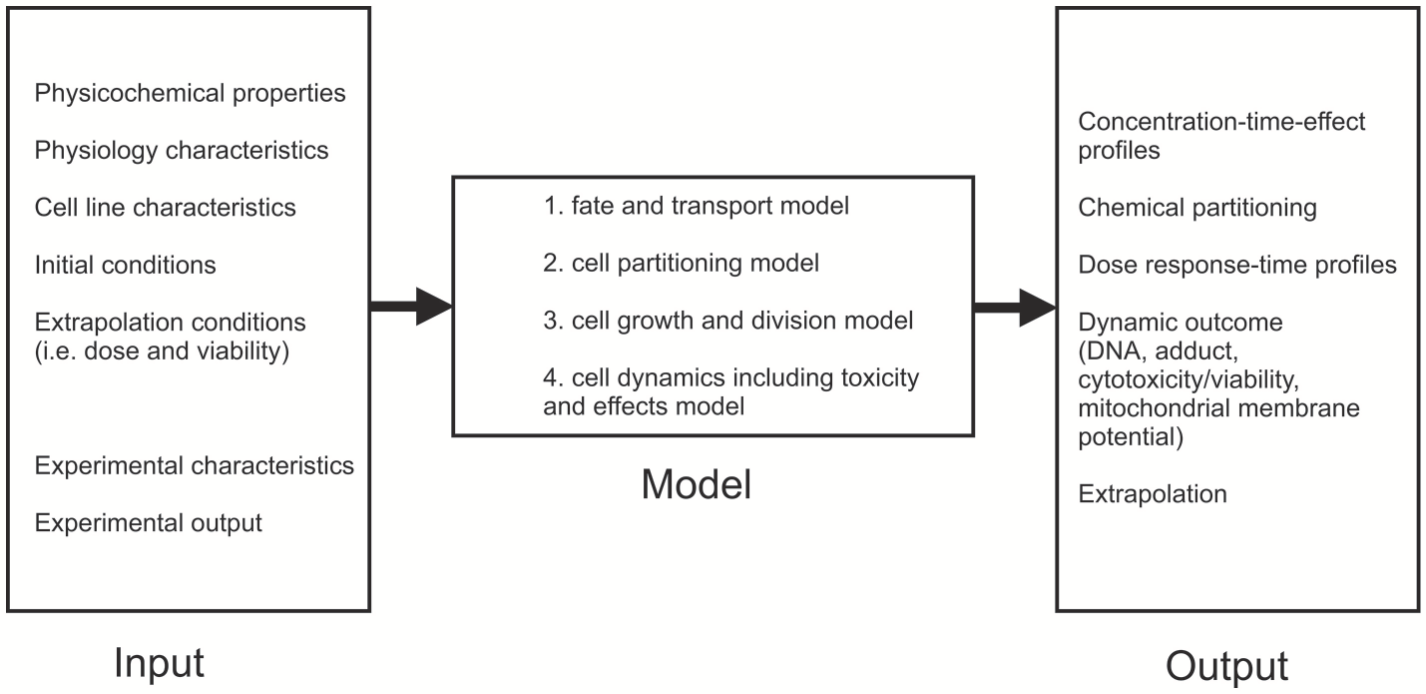
Schematic representation of VCBA model.

Although *in vitro* assays are convenient to estimate toxic mechanisms, there are still some limitations of *in vitro* assays that make it impossible to completely replace *in vivo* studies (Comenges et al., 2017). These limitations mainly concern differences found between *in vitro* and *in vivo* experiments (false positives and negatives, large inter-assay variability, the low sensitivity etc.) (Höfer et al., 2004; Lilienblum et al., 2008). Because of that, mathematical models that comprise the fate of a compound in the cell-based assay combined with a cell growth model are developed (Comenges et al., 2017). Virtual Cell Based Assay (VCBA) was developed from HTS laboratory data (Zaldívar et al., 2010; Zaldívar and Baraibar, 2011) with the aim to describe dynamic effects, since the kinetics is already described by PBK models (Sala Benito et al., 2017). The VCBA can be described as a process-based model or mathematical representation of an *in vitro* assay to simulate the effects and a fate of a chemical (Comenges et al., 2017). The VCBA model (Comenges et al., 2017; Sala Benito et al., 2017) consists of many parameters dependent on the physicochemical, experimental, as well as cell line characteristics, but can be divided into four sub models that describe:

1. Fate and transport model,
2. Cell partitioning model,
3. Cell growth and division model,
4. Cell dynamics including toxicity and effects model (Figure 3).

Fate and transport sub-model calculates time-dependent chemical concentration in the assay, by using dynamic mass balance equations. This means that several phenomena are included (evaporation, absorption onto the plastic, degeneration, partitioning of the chemicals etc.) Further on, cell partitioning sub-model includes extrapolation of the cell model equation, assuming that the total concentration of the compound can be partitioned into the concentrations of the compartments. Cell growth and division sub-model examines four stages G1, S, G2, M of cell cycle, while cell dynamics including toxicity and effects sub-model includes the influence effects of the chemical concentration on the survival rate (by taking into account the mortality rate) (Sala Benito et al., 2017).

Initially, the VCBA was built in Matlab with the aim to examine the toxicological effects of chemicals on cells (Zaldívar et al., 2010; Zaldívar and Baraibar, 2011; Comenges et al., 2012). It was later adapted to R Language, to make it freely available to end-users. Comenges et al. integrated the code into the Knime environment through an R-KNIME node (Comenges et al., 2017). The KNIME Analytics Platform^1^ (Berthold et al., 2009) was developed as a user friendly and free graphical workbench that supports data analytics which includes data management and transformation, investigation, as well as visualization and reporting. In that sense, Benito et al. used the KNIME Analytics Platform, as a user-friendly tool in automation of generating the key input parameters in biokinetic models (Sala Benito et al., 2017).

It should be stated that VCBA models were also used in examining HepG2 cell lines. Paini et al. examined repeated exposure from human liver cell lines HepG2 (and also HepaRG) in order to optimize the VCBA (Paini et al., 2017). The main advantage of this research is that the repeated dose toxicity is not convenient in *in vivo* studies and VCBA models can help reduce and optimize the time spent in the assessment of human safety when it comes to similar research. Additionally, it should be emphasized that the entire projects were dedicated to the development of computational tools and predictive models to estimate the safety of the chemicals. EU COSMOS project^2^ was one of them. Within this project 11 chemical specific PBK models were developed (Bois et al., 2017; Sala Benito et al., 2017). The COSMOS models were inserted in previously mentioned KNIME versions, with two possibilities for execution—locally, on a desktop computer, or remotely, using web browser and KNIME WebPortal^3^.

## On-chip systems

**Figure 4:**
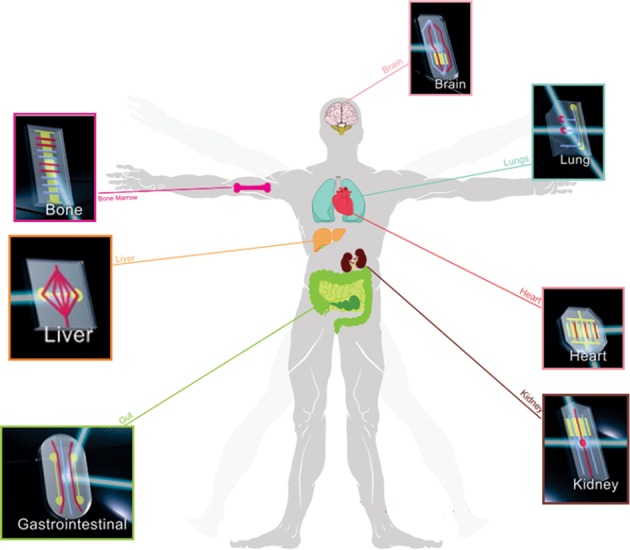
Organs-on-a-chip.

Organ-on-a-chip stands for the microfluidic device that mimics the behavior of a certain organ or system on a microchip. In the past few years, several different organs were developed with on-chip technology (Figure 4).

The major advantage of organ-on-a-chip technology is its capability to represent structural and functional complexity of living tissues and organs, unlike *in vitro* cell culture techniques, which fail in reproduction of dynamic mechanical and biochemical microenvironment. Organ-on-a-chip micro device mimics microsystems and possesses tremendous potential as an innovative and predictive screening platform.

### Lung-on-a-chip

**Figure 5:**
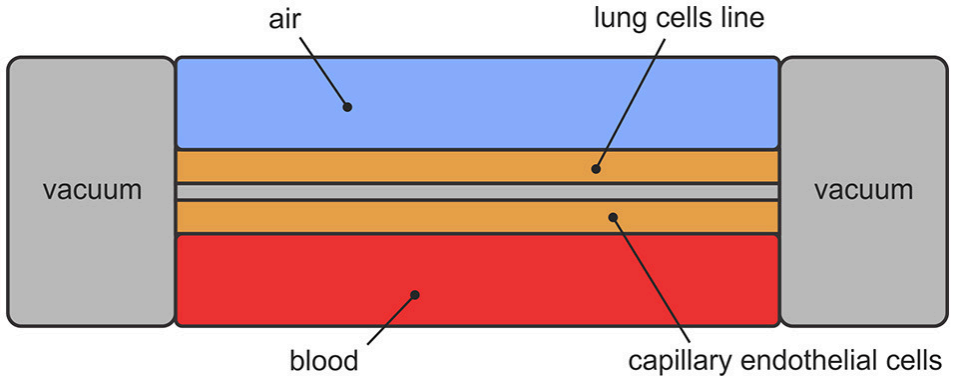
Schematic view of lung-on-a-chip.

Lung-on-a-chip, initially developed at Wyss Institute for Biologically Inspired Engineering at Harvard University, stands for microfluidic device that mimics breathing human lungs on a microchip^4^. The design includes two layers of lung cells separated by porous membrane and covered with two canals—upper and lower. The upper canal represents the airways and permits airflow. The lower canal represents the blood vessels and permits blood flow. Mimicking breathing is achieved with vacuum applied to the chambers, which creates cyclic mechanical stretching. Design of the lung-on-a-chip is presented in Figure 5.

The main purpose of generation of organ-on-chips, including lung, is to reduce and ultimately replace experiments on the animals.

Lung-on-a-chip device can be used to simulate the breathing of a healthy lung and to analyse the interaction of nanoparticles, which derive from air and water pollution and can lead to inflammatory processes. Furthermore, new drugs and therapies for certain lung diseases can be tested, the toxicity of novel drugs can be measured and lung cancer can be simulated.

Huh is one of the founders of lung-on-a-chip technology (Huh, 2015). He also created lung model, focusing on the alveolar-capillary interface—microfluidic device as a unit of living human lung. The model consists of two chambers, separated by porous membrane. The upper chamber contains alveolar epithelial cells and the lower chamber contains pulmonary microvascular endothelial cells. The upper chamber is filled with air and the lower one with blood. Side chambers are used for cyclic vacuum suction to induce cyclic stretching in order to simulate breathing motions and deformation of the alveolar-capillary barrier (Figure 5).

The micro-device has the ability to reproduce lungs' properties. It can be used for better understanding of lungs' function and simulation of pulmonary diseases (Metzger, 2018). Essential processes in breathing lungs are gas exchanges between alveolus and capillary. After established function of gas exchanges, the micro-device can be used for toxicity analysis as well as for analysis of certain pulmonary diseases. This model can be used for analysis of silica nanoparticles absorption and acute toxic response to the nanoparticle absorption due to stretching of alveolar-capillary barrier. This leads to conclusion that breathing increases nanoparticles absorption^5^

In the Wyss laboratory^4^, alveolus-on-a-chip model was developed for reproducing pulmonary thrombosis. To simulate an intravascular thrombosis, the tumor necrosis factor (TNF-α) was added to the upper chamber. The aim was to provoke inflammation involving cytokines, which alert leukocytes and platelets blood particles. These blood particles further trigger thrombi formation. It was confirmed at the Wyss institute that permeability of the lung was related to the TNF-α concentration (Jain et al., 2018). The use of LPS (lipopolysaccharides) induced thrombosis and caused inflammation by interactions between alveolar epithelial and vascular endothelial cells. In conclusion, drug Parmodulins reduced vascular inflammation without endothelial injury. Therefore, one of the potential applications of lung-on-a-chip technology is the analysis of thrombosis. Another one can be the analysis of the physiological reactions during acute asthmatic crisis (Benam et al., 2015). Different types of pulmonary diseases can be analyzed using the micro-device. Novel drug testing for observed disease can be produced without using animals and with reduced costs. Pulmonary edema can be analyzed as well. It is the condition where blood clots and fluid are filling the lung, caused by heart failure or cancer drug side effects. Huh (2015) tested the model presented in Figure 5 to pulmonary edema and the cancer drug was injected into the lower chamber for that purpose. Migration of the fluid and plasma protein into the upper air chamber was noticed, similar to the drug's side effect. The immune system is not involved in leakage side effects, as it was thought. In fact, breathing increases leakage (Rojahn, 2012). Usage of different drug candidates, such as GlaxoSmithKline drug, is found to be able to prevent the leakage in the chip system (Rojahn, 2012). Small airway-on-a-chip was developed for the analysis of lung inflammation and drug responses (Benam et al., 2015). Airway-on-a-chip was created by seeding primary human airway epithelial cells (hAECs) and primary human lung microvascular endothelial cells on the opposite sides of the porous membrane. Epithelial cells formed a barrier by creation of tight junctions, which were detected by ZO1 expression, while endothelial cells junctions were observed by PECAM-1 expression. Measurements of airway-on-a-chip were compared with *in vivo* measurements, in order to validate the usage of micro device. Electron microscopic analysis confirmed that the cilia at the apical side had the same structure as healthy cilia found in living human lung *in vivo* (9 + 2 microtubule organization). Cilia beat actively at a frequency range [9–20 Hz]. Mucociliary transport was measured and it was observed that the particle velocity was nearly identical to the one observed in healthy human lungs' airway. The conclusion is that airway-on-a-chip can be used as a representation of the human living healthy lung and further for asthma and chronic obstructive pulmonary disease (COPD). IL-13 has an important role in asthma evolving and it is sufficient and necessary for induction of allergic asthma in animal models. It participates in airway inflammation, goblet cell hyperplasia and mucus hypersecretion, as well as in creation of subepithelial fibrosis and airway hyper-responsiveness. Increase in the number of goblet cells was noticed after 8 days when small airway-on-a-chip was treated with IL-3, like in other *in vitro* models. Higher production of inflammatory cytokines G-CSF and GM-CSF into vascular channel and decrease in cilia beating frequency were also noticed. Pathogenic infections are major cause of COPD exacerbation in patients. Airways-on-a-chip epithelial cells were lined up with either normal or COPD epithelial cells and treated with the viral mimic poly (I:C) or lipopolysaccharide endotoxin (LPS). Bacterial wall derived component that simulates cytokine production and has been widely used for that purpose. M-CSF cytokine secretion was analyzed, because it promotes differentiation and survival of macrophages. On the other hand, production of the IL-8 was monitored, because IL-8 attracts neutrophils. Stimulation with poly(I:C) promoted secretion of the cytokines IP-10 and RANTES in both healthy and COPD chips. Besides IP-10, which is an excellent clinical marker, level of M-CSF was increased in poly(I:C) treated airway chip. For this reason, it could be used as a new biomarker for COPD exacerbations induced by respiratory viruses. The developed microfluidics models of human asthmatic and COPD airways can be further used for testing the efficacy of new experimental drugs and also for dissection of the mechanism of drug action at the molecular level.

Cells from lung-on-chip micro device can be monitored with microimpedance tomography system, which can provide useful information about the cells and culture conditions (Mermoud et al., 2018). Novel microimpedance tomography (MITO) can be integrated in a lung-on-chip and can allow the real-time monitoring of the integrity of an epithelial barrier located 1 mm away from the electrodes. This system successfully monitored in real-time resistivity changes occurring on the lung epithelial barrier.

Yang et al. developed tumors-on-a-chip with PLGA electrospinning nanofiber membrane, which thickness is about 3 μm and that is porous and permeable to molecules (Yang et al., 2018). Gefitinib drug, an EGFR-targeted anti-tumor drug, was evaluated and A549 cell resistance in the co-culture with HFL1 was analyzed. One of the reasons could be secretion of IGF-1 by HFL1 cells which activated PI3K/Akt signal pathway after inhibition and led to low response of the tumor cells to the chemotherapeutic drugs. Also, A549 cells cause apoptosis and death of the endothelial cells and this can further lead to tumor spreading. The final goal of the authors (Yang et al., 2018) is to apply model in personalized treatment of the lung tumor, which can be important in future clinical research.

### Liver on-a-chip

The main reason for the failure of possible treatments after testing on animals is the hepatotoxicity (Ho et al., 2006; Lee et al., 2015). The possibility of using livers-on-chips enables us to avoid this stage. “Organ-on-a-chip” concept started as a way of mimicking tissues and organs by constructing the networks composed of several connectional functional units connected by multi-channels (Baker, 2011; Bhatia and Ingber, 2014; Esch et al., 2015). However, liver cells are said to be some of the most difficult cell lines for keeping alive in a Petri dish (Domansky et al., 2013) and a way of mimicking their normal environment is of crucial importance in order to increase their lifespan. Parker et al. (2008) showed that liver cells multiplied and their metabolic activity was increased when mesenchymal cells from human placenta were placed together with liver cells. The most important parameter in this process is the ratio between the two aforementioned types of cells. The microfluidic cell line devices are given full attention due to the advantage of small length scale and flow control (Sia and Whitesides, 2003). Some initial attempts toward real life applications of on-chip systems in tissue engineering included examination of impedance measurements in order to determine where certain cell aggregates are placed in a biologically relevant 3D environment (Canali et al., 2015). In normal biological environment, cells are surrounded by hydrogel-like extracellular matrix (ECM) made out of proteins (Canali et al., 2015). When alternating electric field is induced, cells and tissues exhibit complex behavior, which is mainly dependent on the frequency (Yang et al., 1999). This was the main assumption behind the research by Canali et al. (2015) who performed finite element (FE) simulations to examine 4T impedance sensing. They introduced high density of human HepG2 cells encapsulated in gelatine through artificial 3D cell constructs and wanted to determine the sensitivity field distribution depending on the combinations of current carrying (CC) and voltage pick-up (PU) electrodes. Simulations were performed in COMSOL Multiphysics software and showed that this method is suitable for impedance-based sensing with the application on formation of bio artificial organs (Canali et al., 2015).

Micro fabricated devices have been examined with various silicon and poly-dimethylsiloxane (PDMS) substrates in order to use it for hepatocyte culture in membrane-based bioreactors (De Bartolo et al., 2000; Ostrovidov et al., 2004), multi-layer polymer structures (Leclerc et al., 2004), and devices that include oxygen and nutrient delivery systems accompanied by online monitoring (Powers et al., 2002; Lee et al., 2006). Ho et al. (2006) have managed to recreate a liver lobule (functional unit of the liver) on a microfluidic chip using dielectrophoresis. Weng et al. (2017) managed to design a device without a scaffold, primarily because scaffold-based technologies have serious limitations (e.g., inherent stability of a scaffold or unpredictable effects on signaling pathways).

A very important already mentioned aspect of on-chip systems is the appropriate distribution of nutrients where non-uniform distribution and shear stresses throughout the scaffold region can decrease cell colonization and influence the quality of the regenerated tissue (Podichetty et al., 2015). For that purpose, axial-flow bioreactors (also named perfusion bioreactors) have been examined to determine optimal flow rates (Gardel et al., 2013), inlet and outlet diameters, as well as scaffold surface for hepatocyte cultivation (Leclerc et al., 2004). The purposes of aforementioned researches were said to be found in bone regeneration (Azuaje, 2010), cardiac patch (Dvir et al., 2006), and abdominal wall (Pu et al., 2010). Some modeling studies used computational fluid dynamics (CFD) to gain insight into fluid dynamics and nutrient distribution in bioreactors (Cioffi et al., 2006; Wendt et al., 2008; Patrachari et al., 2012). They used either a multi-scale continuum modeling approach (Causin et al., 2013) or Boltzmann-Lattice model (Spencer et al., 2013). Their approach using modeling is useful, but the main drawback in the research is the lack of experimental validation (Podichetty et al., 2015). They examined nutrient distribution characteristics because of the influence of the non-ideal flow on tissue regeneration (Podichetty et al., 2015). The results of the CFD simulation and dispersion model were validated with experiments of nutrient consumption (Podichetty et al., 2015). Metabolically very active HepG2 cells were seeded on chitosan-gelatin (CG) scaffolds and the results showed that *in vitro* tissue regeneration monitoring is possible with this kind of simulation. This represents a step forward toward the understanding the mechanical stimulus effect on 3D cell culture (Podichetty et al., 2015).

**Figure 6:**
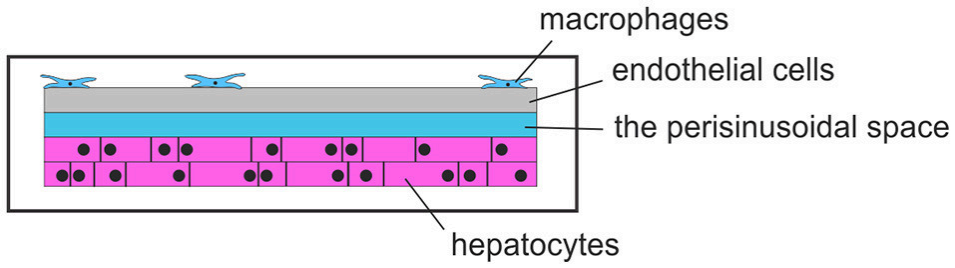
Schematic view of liver-on-a-chip.

A research group at the Universitätsklinikum Jena, Germany, developed liver-on-chip^6^. They examined the activation patterns of macrophages in inflammatory responses, in order to control bacterial infections. A liver-on-chip model with microfluidic perfusions was made to examine liver infections. With all essential cell types of the human liver on this chip and emulation of a microphysiological environment, this chip can be used to examine inflammation-associated molecular processes of hepatic function disorder and macrophage-associated processes of tissue repair that is subjected to the inflammatory conditions. Particularly, this research group used the described liver-on-chip system to investigate the pathogenesis of *Staphylococcus aureus* infections and infections with *Candida albicans*, as well as in treatment of sepsis-associated hepatic disorders by using functionalized nanoparticles. A schematic view of a liver-on-a-chip is given in Figure 6.

A multi-type cell on-hip model was made by Jie et al. (2016) to reconstruct physiological drug kinetics by seeding Caco-2, HepG2, and U251 cells (they were used to mimic the intestine, liver, and glioblastoma, respectively). A full description of the microfluidic on-chip was given by Jie et al. (2016), where Caco-2 cells served as intestine tissue for oral drug absorption. HepG2 cells, as a liver equivalent, were placed below the Caco-2 cell line. U251 cells, as a glioblastoma compartment, were connected with HepG2 cells unit by narrow channels array (Jie et al., 2016). All this could be said to mimic *in vivo* 3D microenvironment and examine the functions of drug absorption, metabolism and response in a produced microenvironment, with the possible application in personalized cancer therapy (Jie et al., 2016). Other researchers also examined microfluidic chips (Huh et al., 2012; Ramadan et al., 2013). Imura et al. (2013) simulated distribution of drugs and excretion processes in a dynamical microfluidic system, while Maschmeyer et al. (2015) established a microphysiological multi-organ system for long-time co-cultivation. The study by Zhang et al. (2008) also needs to be mentioned, as this research specifically deals with microfluidic environment, designed for improved hepatocyte liver cell culture *in vitro*. Finite element simulations modeled using COMSOL were performed in order to examine the effect of continuous perfusion on glucose consumption by HepG2 cell (Zhang et al., 2008). The results show that the suggested method is appropriate for production of microfluidic devices with mass transport conditions, as well as discuss the effects on maintenance of biological function and differentiated phenotype (Zhang et al., 2008).

Additionally, designs that consist of interconnected compartments allow research on how different cell types and organs communicate and interact in the sense of interdependent cellular responses. Theobald et al. examined simplified liver-kidney-on-chip model and concluded that the combined liver-kidney model is adequate for initial determination of liver mediated toxic effects. They showed that hepatic cells, growing in large amounts in microfluidic conditions, expressed metabolism-related biomarkers (Theobald et al., 2017). Investigation of toxicity of Aflatoxin B1 (AFB1) and Benzoalphapyrene (BαP) showed the importance of examining multi-organs in microfluidic devices for the purposes of *in vitro* toxicity testing and drug screening (Theobald et al., 2017). Chang et al. developed an integrated liver-kidney microphysiological (MPS) system to identify nephrotoxic liver-metabolized chemicals using the connected liver-on-a-chip and kidney-on-a-chip (Chang et al., 2016). Their results show the important specific purpose of hepatic biotransformation in toxicity research and validation of *in vitro* and *in vivo* model comparisons (Chang et al., 2016).

## Conclusions

Many scientists are skeptical when it comes to further improvement of the organ-on-a-chip systems and replacement of animal experiments because human organism is very complex by structure and function, and they do not believe that organ-on-a-chip can include all the environmental parameters. That is true—lung-on-a-chip is not a real lung, but there can be certain benefits from finding more details related to specific lung diseases. There can be benefits from testing the novel drugs before trying them on animals. Experiments on animals should fill ethical requirements and cannot be related to the human lungs in every aspect (Benam et al., 2015). For example, mouse and rat lungs have less frequent mucin-producing cells, which are important for asthma developing. Lung inflammation involves complex tissue-tissue interactions between lung airway epithelium cells and microvascular endothelium cells that activate the immune system—recruit immune cells, such as neutrophils. These processes can be simulated by using lung-on-a-chip microfluidic device. Besides regular behavior of the lung, these microfluidic devices can be used for collecting information and new knowledge about lung diseases, as well as for testing novel drugs approaches and treatments.

As far as liver cell line studies are concerned, it was reported that drug-induced hepatic injury in liver (liver hepatotoxicity) is the most common reason for delays in the clinical phase in drug development and even withdrawal of an approved drug (Bies et al., 2006). Contrary to other human organs, liver has a remarkable capacity to regenerate. Along with the physiological importance and plasticity of this organ, a better understanding of human physiology, disease and response to exogenous compounds is enabled through the examination of the liver. Additionally, the main challenge for the construction of microfluidic liver devices is the complexity of the liver itself. Due to its complexity and numerous functions combined with different types of cells and a specific construction, the liver is very hard to model. Therefore, the main convenience of the liver-on-a-chip is its ability to replicate microscopic details, which is important in future examination (Lee et al., 2015). Once the improvements in liver-on-a-chips are made, curing a lot of diseases (e.g., hepatitis B, hepatocellular carcinoma) will become easier.

## Author contributions

All authors listed have made a substantial, direct and intellectual contribution to the work, and approved it for publication.

### Conflict of interest statement

The authors declare that the research was conducted in the absence of any commercial or financial relationships that could be construed as a potential conflict of interest.

## Footnotes

^1^Available online at http://www.knime.org

^2^Available online at http://www.cosmostox.eu/home/welcome

^3^Available online at https://knimewebportal.cosmostox.eu/com.knime.enterprise.server

^4^Wyss Institute. (November 07, 2012). Available online at: https://wyss.harvard.edu/wyss-institute-models-a-human-disease-in-an-organ-on-a-chip.

^5^Wyss Institute. (June 24, 2010). Available online at https://wyss.harvard.edu/living-breathing-human-lung-on-a-chip-a-potential-drug-testing-alternative/.

^6^Available online at https://www.uniklinikum-jena.de/cscc/mosig.html (Accessed May 05, 2018)
